# In vitro characterization of CD133^lo^ cancer stem cells in Retinoblastoma Y79 cell line

**DOI:** 10.1186/s12885-017-3750-2

**Published:** 2017-11-21

**Authors:** Rohini M. Nair, Murali MS. Balla, Imran Khan, Ravi Kiran Reddy Kalathur, Paturu Kondaiah, Geeta K. Vemuganti

**Affiliations:** 1 School of Medical Sciences, University of Hyderabad, Hyderabad, 500046 India; 2 Ophthalmic Pathology Laboratory, L V Prasad Eye Institute, Hyderabad, 500034 India; 3 Radiation Signalling and Cancer Biology Section, RB & HSD, BSG, Bhabha Atomic Research Centre, Mumbai, 400085 India; 4 Molecular Reproduction, Development and Genetics, Indian Institute of Science, Bangalore, 560012 India; 5 National Cancer Institute, National Institute of Health (NIH), Bethesda, MD 20892 USA; 6 Department of Biomedicine, University of Basel, 4058 Basel, Switzerland

**Keywords:** Retinoblastoma, Stem-like cancer cells, Cancer stem cell markers, CD133 (Prominin), Flow cytometry

## Abstract

**Background:**

Retinoblastoma (Rb), the most common childhood intraocular malignant tumor, is reported to have cancer stem cells (CSCs) similar to other tumors. Our previous investigation in primary tumors identified the small sized cells with low CD133 (Prominin-1) and high CD44 (Hyaluronic acid receptor) expression to be putative Rb CSCs using flow cytometry (FSC^lo^/SSC^lo^/CD133^lo^/CD44^hi^). With this preliminary data, we have now utilized a comprehensive approach of in vitro characterization of Y79 Rb cell line following CSC enrichment using CD133 surface marker and subsequent validation to confirm the functional properties of CSCs.

**Methods:**

The cultured Rb Y79 cells were evaluated for surface markers by flow cytometry and CD133 sorted cells (CD133^lo^/CD133^hi^) were compared for CSC characteristics by size/percentage, cell cycle assay, colony formation assay, differentiation, Matrigel transwell invasion assay, cytotoxicity assay, gene expression using microarray and validation by semi-quantitative PCR.

**Results:**

Rb Y79 cell line shared the profile (CD133, CD90, CXCR4 and ABCB1) of primary tumors except for CD44 expression. The CD133^lo^ cells (16.1 ± 0.2%) were FSC^lo^/SSC^lo^, predominantly within the G0/G1 phase, formed larger and higher number of colonies with ability to differentiate to CD133^hi^ cells, exhibited increased invasive potential in a matrigel transwell assay (*p* < 0.05) and were resistant to Carboplatin treatment (*p* < 0.001) as compared to CD133^hi^ cells. The CD133^lo^ cells showed higher expression of several embryonic stem cell genes (*HOXB2, HOXA9, SALL1, NANOG, OCT4, LEFTY*), stem cells/progenitor genes (*MSI2, BMI1, PROX1, ABCB1, ABCB5, ABCG2*), and metastasis related gene- *MACC1*, when compared to the CD133^hi^ cells.

**Conclusions:**

This study validates the observation from our earlier primary tumor study that CSC properties in Rb Y79 cell line are endowed within the CD133^lo^ population, evident by their characteristics- i.e. small sized, dormant in nature, increased colony forming ability, differentiation to CD133^hi^ cells, higher invasiveness potential, drug resistance and primitive gene expression pattern. These findings provide a proof of concept for methodological characterization of the retinoblastoma CSCs with future implications for improved diagnostic and treatment strategies.

## Background

Retinoblastoma is the most common paediatric ocular malignant tumor occurring in 1 of every 15,000–20,000 live births [1, 2]. This tumor is caused due to inactivation of both the alleles of Retinoblastoma (*RB1*) gene resulting in the defective pRB protein. *RB1* is a major tumor suppressor gene that is involved in cell cycle progression, DNA replication and terminal differentiation [3]. Loss of pRB activity in the retinal progenitor cells leads to impaired cell cycle, uncontrolled cell proliferation and tumor progression. In addition to RB1 as the rate-limiting step for tumor initiation, there are multiple genes (oncogenes and tumor suppressor genes) that undergo mutations, such as *MYCN* gain, loss of 16q, etc., thereby promoting tumorigenesis [4, 5]. Recent studies have shown that there are cases of unilateral Retinoblastoma that are devoid of Rb mutations and these tumors have distinct histological and genomic landscapes (e.g. high MYCN expression) that facilitate aggressive tumor formation similar to that seen in RB1(−/−) tumors [4, 6]. The hypothesis of cancer stem cells (CSCs), which is now synonymous with tumor initiating cells (TICs) and stem-like cancer cells (SLCCs), originated first from blood related cancers, wherein a small fraction of the tumor cells were reported to be responsible for tumor formation and were attributed with properties of normal stem cells such as quiescence, proliferation, and drug resistance [7]. The salient features of both CSCs and normal stem cells are their potency for self-renewal and forming a cellular hierarchy within the tumor/normal tissue. Additionally, both stem cells and CSCs have the ability to differentiate and migrate [8]. In paediatric brain cancers, tumor derived progenitors form neurospheres that can be passaged at clonal density and are able to self-renew. These cells express several genes characteristic of neural and other stem cells including *CD133, NESTIN, SOX2, MSI1, BMI1, MELK, OCT4*, etc. [9, 10].

In retinoblastoma, several studies have reported the presence of stem cells in both primary tumors as well as cell lines, using a few properties attributed to cancer stem cell phenotype, ability to actively extrude drugs, slow cycling, clone formation post nutrient starvation, etc. [11–16]. Seigel and co-workers showed the presence of Hoechst dye exclusion, Bromodeoxyuridine (BrdU) label retaining cells, Aldehyde Dehydrogenase 1 (ALDH1) and stem cell markers in the total population of Rb cell lines, primary tumors and simian virus-40 luteinising hormone β sub-unit Large T-antigen (SV40 βLH-T-Ag) mouse tumors [12, 13]. In cultured primary tumors cells, expression of a few retinal development related genes and in vivo tumorigenicity was demonstrated by Zhong et al., thereby hinting at a presence of stem-like cancer cells within Rb tumors [17]. Our group provided evidence of putative stem-like cells in primary Rb tumor cells using a bi-parameter model by flow cytometry with a phenotype of low CD133, high CD44 expression and small sized cells (FSC^lo^/SSC^lo^/CD133^lo^/CD44^hi^) expressing progenitor cell markers (*PROX1* and *SYX1A*) [18]. Similar to our study on primary Rb, we observed the two parametric distribution of Rb Y79 cell line with a small subset of cells exhibiting low forward and side scatter profile with low CD133 expression (FSC^lo^/SSC^lo^/CD133^lo^). It is interesting to note that other than the studies on normal developing retina and Rb deficient retinal cells, which suggests that CD133 expression is low in progenitors and high in differentiated photoreceptors [19, 20], the Rb tumor studies suggest that CD133 expression is specific to cancer stem cells [15, 17, 21]. This prompted us to investigate the surface marker profile of Y79 cell line and compare the functional properties of CSCs [22] within the CD133 sorted populations.

## Methods

### Cell culture

Retinoblastoma Y79 cells (Riken: RCB1645 Y79 - a generous gift from Dr. S. Krishnakumar, Sankara Nethralaya, Chennai, India) were revived and cultured in Roswell Park Memorial Institute-1640 (RPMI-1640) media supplemented with 10% Fetal Bovine Serum (FBS), antibiotics and L-Glutamine (Gibco™, ThermoFisher Scientific). The cell line was authenticated at the time of purchase from Riken. Regular mycoplasma tests have been carried out and the experiments were conducted in compliance with good laboratory practices (GLP). Media was changed every 3 days and the cells were sub-cultured following observation of cell confluency of about 70%. Enrichment and characterization of putative cancer stem cells were carried out in the cultured Y79 cells and then sorted using the surface marker, CD133. In vitro functional characterization of the sorted subsets was carried out to assess the cell cycle status, clone forming ability, differentiation, invasion, chemoresistance and gene expression signature.

### Flow Cytometry analysis and sorting

One million Y79 cells were stained by incubating with directly labelled primary antibodies (CD133-Miltenyi Biotech, CD44, CD90, CXCR4-Ebioscience and ABCB1-Abcam) for 45 min at 4 °C. The antibodies were standardized by varying their dilutions and checking the expression percentage. The cells were then washed thrice with wash buffer to remove excess antibody and run in the BD LSRFortessa™ flow cytometry analyser and the analysis was done using FACSDiva™ software version 6.2. Appropriate controls were used for the experiments. A total of 20,000 to 50,000 events were acquired for analysis. The cells were gated based on size, granularity and doublet discrimination as described previously [18]. In brief, Y79 cells were first selected based on forward and side scatter, and the doublets were excluded using the doublet discrimination plots. The negative control (unstained cells) is used to set the laser voltages, sorting gates and establish the Allophycocyanin (APC) expression profile for the population. The labeled cells were then run through the cytometer and the two populations (CD133hi and lo) are collected in tubes with medium containing 2X antibiotic solution. The post-sort purity and viability was determined. The sorted cells were then used for CSC characterization.

### Magnetic activated cell sorting

The Y79 cells in their growth phase were sorted using the CD133 Microbead Kit according to the manufacturer’s protocol (Miltenyi Biotec Inc., Auburn, CA). Briefly, the cells were centrifuged at 300 g for 10 min and resuspended in 300 μL of buffer per 10^7^cells. 100 μL of FcR Blocking Reagent was added and mixed well. To the cell suspension, 100 μL of CD133 Microbeads were added, mixed well and incubated for 30 min at 4 °C. The cells were washed twice with the buffer and resuspended in 500 μL of buffer. Magnetic separation was carried out in LS columns using the MiniMACS™ separator and the two populations were collected in labelled tubes. The sorted cells were then assessed for CD133 expression using flow cytometry for determining the sorting purity and further experiments were carried out following viability count using Trypan blue.

### Cell cycle analysis

Sorted CD133^hi^, CD133^lo^ and unsorted total Y79 cell populations were pelleted by centrifugation and resuspended in PBS with 50 μg/ml propidium iodide for cell cycle analysis. After incubation on ice for 30 min, cell populations were treated with 0.25 mg/ml RNaseA for 45 min at 37 °C to remove RNA. Cells were analysed by flow cytometry at an excitation wavelength of 488 nm and the cell cycle histogram was assessed using the BD FACSDiva software.

### Soft agar Colony formation assay and differentiation

The sorted CD133^hi^ and CD133^lo^ Y79 cells were grown in agarose as single cells to assess their colony forming potential. Briefly, a base coat of 0.8% agarose was added into the wells of a 24-well plate and further covered with cell suspension (1000 cells/well in 0.48% agarose). Plates were incubated for 2 weeks following which the resulting colonies were then fixed with 3.7% paraformaldehyde and stained with crystal violet. The images of the colonies were taken at 1.5X and 4X magnification and analysed using ImageJ and OpenCFU software. For colony forming efficiency (CFE) analysis, colonies greater than 50 cells were counted and percentage of colonies were calculated. The morphology of the colonies was assessed for characteristics of holoclones, meroclones and paraclones. The CD133^lo^ clones were then expanded and the expression of CD133 was checked for four passages in vitro.

### Matrigel Transwell invasion assay

The Matrigel transwell invasion assays were performed using Corning transwell 24-well inserts with 8 μm pore size as per manufacturer’s instructions. Briefly, 10^4^ cells each of CD133^hi^ and CD133^lo^ subsets were serum-starved overnight and plated into 100 μl serum-free medium onto the inner chamber of the transwell plate, which was previously coated with Matrigel (1:50) (BD Biosciences). The bottom well was then filled with 600 μl of media with 10% serum. The cells were incubated for 24 h at 37 °C and 5% CO_2_ level. Following incubation, the media was removed from the plate and the non-invasive cells were scraped off from the upper side of the insert and the cells in the lower side of the membrane were fixed in 3.7% Paraformaldehyde (PFA), washed, and stained using crystal violet. Experiments were performed in triplicate transwell and the invaded cells were quantified by counting the average number of cells per 20X field of view and 10 fields per chamber.

### Cytotoxicity assay

The sorted Y79 cells (CD133^hi^ and CD133^lo^) were assessed for chemoresistance against Carboplatin (Alkem Pharmaceuticals) using MTT assay. Briefly, 5000 cells/90 μL media each of the populations were seeded in a 96 well plate and incubated overnight at 37 °C and 5% CO_2_ level. Carboplatin was added at varying concentrations (1 μM–100 μM) at a final volume of 10 μL in the wells and incubated for 48 h. Following incubation, 20 μL of 5 mg/mL MTT reagent was added to each well and incubated for 3 h. The Formazan crystals formed were dissolved in 100 μL of Dimethyl sulfoxide (DMSO) and the absorbance was recorded at 595 nm using an ELISA plate reader. The percentage of viability was calculated compared to the controls for each of the population and drug concentration.

### Gene expression microarray and pathway analysis

Microarray was performed in duplicates using human whole genome (4x44K) cDNA arrays (Agilent technologies, USA). Labelling reactions were performed using 500 ng of RNA from CD133^hi^ and CD133^lo^ populations. Labelling of the probes was carried out using the Low RNA input linear amplification kit (Agilent technologies, USA) where total RNA was first converted to cDNA using T7-oligo d(T) primers. From this cDNA, labelled cRNA was generated via an in vitro transcription reaction using T7 RNA polymerase and Cy3 (for CD133^hi^ population) or Cy5 (CD133^lo^ population) labelled CTP respectively. Probes with higher labelling efficiency (specific activity ≥8 pmol Cy3 or Cy5/ng cRNA) were selected for competitive hybridization as per the manufacturer’s instructions. 825 ng each of Cy5 and Cy3 labelled cRNAs from CD133^lo^ and CD133^hi^ populations were mixed and added to hybridization buffer and placed on the array. Hybridization was done in a chamber (Agilent technologies, USA) for 17 h at 65 °C with gentle rotation. The slide was scanned and image was analysed using feature extraction tool version 9.5.3.1 (Agilent technologies, USA) and data was analysed using GeneSpring version 10 (Agilent technologies, USA). Lowess algorithm was used to normalize the data. Fold change was calculated based on ratio of Cy5/Cy3 intensities and genes with fold change ≥ + 1.5 or ≤ − 1.5 where considered differentially regulated for which with *p*-values were assessed. Further, we performed functional enrichment analysis to identify enriched biological processes and pathways in our differentially regulated genes using DAVID bioinformatics resources (version 6.8) and KEGG pathway database [23, 24]. Biological processes and pathways with *p*-value ≤0.05 where considered significantly enriched. Additionally, we validated the up-regulated genes involved in pathways using Polymerase Chain Reaction (PCR).

### Semi quantitative PCR

Total RNA was isolated from the sorted populations by the TRIzol™ method of solubilisation and extraction. The isolated RNA was quantified using Nanodrop and cDNA was prepared using SuperScript™ First-Strand Synthesis System kit (Invitrogen). The prepared cDNA was then analysed for the expression of *ACTB, BMI1, CD133, NANOG, PROX1, MACC1, SNAI2* and *ABCG2* genes by semi-quantitative PCR. The primer sequences used for PCR are enlisted in Table 1. The samples were then observed for gene expression using a 2% agarose gel and the image was captured using BioRAD ChemiDoc™ and Image Lab software.

**Table 1:** Primer sequences for the genes used in semi-quantitative PCR

S No.	Gene	Forward primer	Reverse primer
1.	ACTB	atgcagaaggagatcactgc	tcatagtccgcctagaagca
2.	CD133	cctctggtggggtatttctt	aggtgctgttcatgttctcc
3.	BMI1	gcttcaagatggccgcttg	ttctcgttgttcgatgcatttc
4.	NANOG	caaccagacccagaacatcc	ttccaaagcagcctccaag
5.	OCT4	atgcattcaaactgaggtgcctgc	ccaccctttgtgttcccaattcct
6.	PROX1	caagttgtggacactgtggt	gcagactggtcagaggagtt
7.	MACC1	cggtcaggaagaattgcac	ttaccacgaagggtgaaagc
8.	SNAI2	tgtgacaaggaatatgtgagcc	tgagccctcagatttgacctg
9.	ABCG2	ggaactcagtttatccgtgg	cgaggctgatgaatggagaag

### Statistical analysis

The quantitative data were stated as Mean ± SEM, and GraphPad Prism (GraphPad Software, La Jolla, CA) was used for unpaired Student’s t-test and ANOVA with Bonferroni’s Post-hoc tests. The representative images were analysed using ImageJ software. The experiments were repeated at least thrice with biological replicates and *p* < 0.05 was considered for statistical significant difference between the groups.

## Results

### Phenotypic characterization of Y79 cells and CD133 cell sorting

**Fig. 1:**
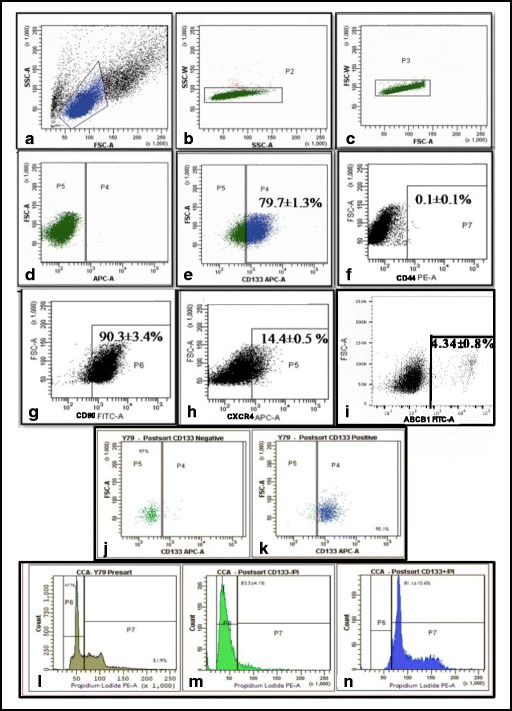
Surface marker expression in Rb Y79 cell line using flow cytometry. Gating of single cells and voltage channel setting- (**a**) FSC vs SSC plot, (**b**) and (**c**) Doublet discrimination plots (**d**) Unstained control. Analysis of various surface markers with representative distribution of various sub-populations within the cell line (**e**) CD133-APC (**f**) CD44-PE (**g**) CD90-FITC, (**h**) CXCR4-APC (**i**) ABCB1-FITC. **j** and (**k**) Purity of sorted CD133^lo^ and CD133^hi^ cells was recorded as >90%. Cell cycle analysis of the total Y79 cells, CD133^lo^ and CD133^hi^ subsets highlighting the G0/G1 status of CD133^lo^ cells (**l**) Cell cycle distribution of total Y79 cells (**m**) CD133^lo^ cells predominantly observed in the G0/G1 phase (83.3 ± 4.1%) (**n**) CD133^hi^ cells mainly observed in the proliferating S/G2/M phase (81.1 ± 4.1%)

Surface marker analysis was carried out in Y79 cell line to analyse the putative CSC markers similar to those observed in primary Rb tissues. The flow cytometry analysis showed the expression of the surface markers on Y79 as depicted in Fig. 1. CD133, CD90, CXCR4, CD44 and ABCB1 constitute 83.25 ± 0.85, 79.7 ± 1.3%, 14.4 ± 0.5%, 0.1 ± 0.1%, and 4.34 ± 0.8% respectively (Fig. 1a-i). The sorting purity of CD133^lo^ and CD133^hi^ was obtained as ≥90% (Fig. 1j, k). Cell viability for the populations was found to be 86.88 ± 3.89% and 87 ± 2.79% respectively.

### CD133^lo^ cells are predominantly in the resting phase of cell cycle

The cell cycle status was assessed in the two populations using flow cytometry to compare the dormant and proliferative compartments. Cell cycle analysis revealed that the majority of the CD133^lo^ population segregated with resting phase i.e. G0/G1 (83.3 ± 4.1%). On the contrary, 81.1 ± 4.1% of CD133^hi^ cells were in S/G2/M phase as shown in Fig. 1l-n, suggesting that CD133^hi^ cells are mitotically active when compared to dormant CD133^lo^ cells.

### CD133^lo^ cells are clonal in nature and differentiate to CD133^hi^ cells in vitro

**Fig. 2:**
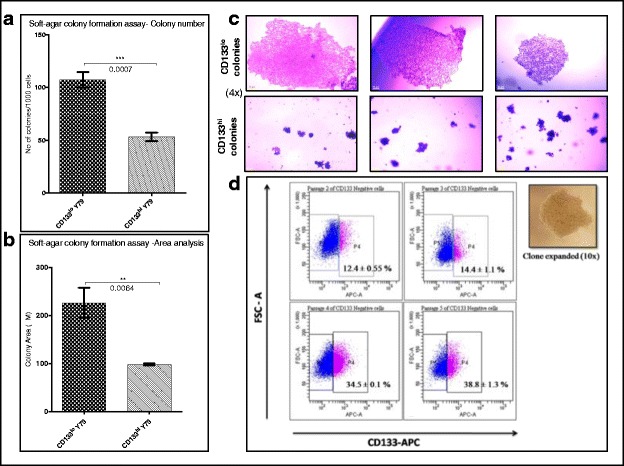
CD133^lo^ cells exhibited clonogenicity and differentiation. **a** CD133^lo^ cells generated increased number of colonies when compared to CD133^hi^ cells (*p* = 0.0007) (**b**) CD133^lo^ colonies had larger area when compared to the CD133^hi^ cells (*p* = 0.0064) (**c**) Representative brightfield images of CD133^lo^ and CD133^hi^ colonies at 4X magnification. **d** CD133 expression profile of expanded CD133^lo^ clone at Passages 2,3,4 and 5 showing differentiation to CD133 expressing cells

In order to assess the clonal nature and differentiation ability of the CSCs, soft agar clonal assays and further expansion was carried out. In the soft agar assays, after 2 weeks of culture, CD133^lo^ cells formed larger number of colonies (<50 cells) when compared to CD133^hi^ cells (107.3 ± 7.4 vs 53.25 ± 3.9, *p* = 0.0007) (Fig. 2a). Colonies formed by CD133^lo^ cells were larger in area when compared to the CD133^hi^ subset (226.0 ± 31.2vs 98.06 ± 1.862, *p* = 0.0064) (Fig. 2b). The colonies of CD133^lo^ cells were compact clusters of uniformly small cells while the CD133^hi^ cell colonies were irregular, with loosely packed large cells (Fig. 2c). Serial passaging of the CD133^lo^ colonies revealed increased level of CD133 expression by passage 4 and 5 (34.5 ± 0.1 to 38.8 ± 1.3%), as shown in Fig. 2d.

### CD133^lo^ cells display increased invasive ability when compared to CD133^hi^ cells

**Fig. 3:**
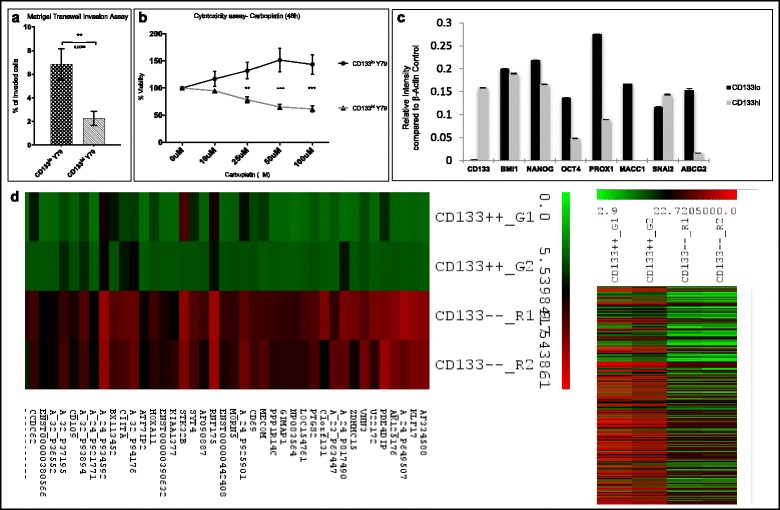
CD133^lo^ cells exhibited invasive ability, resistance to Carboplatin treatment and CSC based gene expression signature. a) Graphical representation of the Matrigel Transwell Invasion assay showing the average number of invaded cells in both populations in response to a chemoattractant (*p* < 0.05). b) CD133^lo^ cells were observed to be more resistant to Carboplatin treatment when compared to CD133^hi^ cells following 48 h exposure (p < 0.05). c) Gene signature of CD133^lo^ cells compared to CD133^hi^ cells with differential expression of stem cell, progenitor, invasion and chemoresistance related genes (p < 0.05) d) Heat map generated for the genes in CD133^lo^ population deregulated by 2-fold change compared to CD133^hi^ cells and hierarchical clustering of CD133lo and hi populations

We evaluated the invasive potential of the two populations through a basement matrix in response to a chemoattractant. Matrigel Transwell assay using 10% serum as chemoattractant for 24 h showed that CD133^lo^ cells exhibited higher invasive potential when compared to the CD133^hi^ cells (6.83 ± 1.3% vs 2.26 ± 0.58% cells per field, *p* < 0.05) (Fig. 3a).

### CD133^lo^ cells exhibit increased chemoresistance to Carboplatin treatment

MTT assay was carried out to assess cytotoxicity of Carboplatin on CD133 sorted Y79 cells following 48 h exposure. CD133^lo^ cells were observed to be more resistant to Carboplatin treatment when compared to CD133^hi^ cells following 48 h exposure (*p* < 0.001) indicating the phenomenon of chemoresistance in this population (Fig. 3b).

### Gene expression microarray and pathway analysis of CD133^lo^ vs CD133^hi^ cells

Comparative gene expression analysis was carried out in the two populations of Y79 cell line to analyse the differentially regulated genes. The top 30 up-regulated and down-regulated genes with fold change ≥ + 1.5 or ≤ − 1.5 are listed in Tables 2 and 3. The gene expression analysis of CD133^lo^ cells, in comparison to CD133^hi^ cells, had an up-regulation of 2945 genes (≥1.5 fold) and down-regulation of 4531 genes (≤ 1.5 fold). The heat map generated for the deregulated genes along with the hierarchical clustering of CD133lo and hi populations are represented in Fig. 3d. Through functional enrichment analysis, we identified Purine metabolism pathway (*p* = 0.009), TGF-β signalling pathway (*p* = 0.009), p53 signalling pathway (*p* = 0.017), Jak-Stat signalling pathway (*p* = 0.047), cytokine-cytokine receptor interaction pathway (*p* = 0.034), and oxidative phosphorylation pathway (*p* = 0.012) to be significantly over-expressed in the CD133^lo^ subset. The embryonic and neural stem cell genes up-regulated (fold change <1.5) in CD133^lo^ population were *HOXB2*, *HOXA9*, *SALL1*, *LEFTY*, *ABCB1*, *ABCB5*, *MUSHASHI2*, *BMI1* (Table 4). Comparative analysis of several stem cell, progenitor, invasion and chemoresistance related genes was further carried out using Reverse transcriptase PCR. *BMI1, OCT4, NANOG, PROX1, MACC1,* and *ABCG2* were observed to be up-regulated in the CD133^lo^ cells when compared to CD133^hi^ cells (Fig. 3c).

**Table 2:** Deregulated genes in CD133^lo^ CSCs when compared to CD133^hi^ non-CSCs. List of top 30 genes up-regulated in CD133^lo^ cells

Gene ID	Gene Name	PValueLogRatio	MEAN_LOG2_R\G_RATIO
AF334588	*Homo sapiens* P25 mRNA	1.25E-16	4.041897
KLF17	Homo sapiens Kruppel like factor 17	3.17E-17	4.037766
A_24_P649507	hypothetical protein MGC5566	1.12E-18	3.992264
AK125176	cDNA FLJ43186 fis, clone FCBBF3022767.	5.17E-16	3.863978
PDE4DIP	phosphodiesterase 4D interacting protein [Homo sapiens (human)]	1.05E-10	3.443785
U22172	Human DNA damage repair and recombination protein RAD52 pseudogene mRNA, partial cds	5.80E-16	3.371156
VNN3	vanin 3 [Homo sapiens (human)]	4.99E-08	3.134369
ZDHHC15	zinc finger DHHC-type containing 15 [Homo sapiens (human)]	4.15E-15	3.10141
A_24_P817490	RST23879 Athersys RAGE Library Homo sapiens cDNA	8.75E-17	3.094543
A_23_P63447	unknown	8.78E-06	3.034319
C1orf131	chromosome 1 open reading frame 131 [Homo sapiens (human)]	3.60E-16	3.004795
PTGS2	prostaglandin-endoperoxide synthase 2 [Homo sapiens (human)]	2.08E-09	2.986919
LOC154761	family with sequence similarity 115, member C pseudogene [Homo sapiens (human)]	6.41E-10	2.874717
NP083564	uncharacterized protein LOC100041774	2.20E-08	2.833521
GIMAP1	GTPase IMAP family member 1 [Homo sapiens]	2.47E-06	2.786683
PPP1R14C	alternative protein PPP1R14C [Homo sapiens]	2.21E-08	2.736343
MECOM	MDS1 and EVI1 complex locus [Homo sapiens (human)]	3.30E-07	2.656235
CD69	CD69 molecule [Homo sapiens (human)]	1.23E-05	2.593014
A_24_P925901	Homo sapiens mRNA for hSSH-2, complete cds. [AB072358]	4.37E-07	2.589578
MORN5	MORN repeat containing 5 [Homo sapiens (human)]	1.41E-05	2.525711
ENST00000442408	ens|cDNA FLJ37906 fis, clone COLON2004318 [Source:UniProtKB/TrEMBL;Acc:Q8N9A9] [ENST00000442408]	9.55E-07	2.521708
RNF175	ring finger protein 175 [Homo sapiens (human)]	2.12E-12	2.477196
AF090887	FLI_CDNA	6.31E-08	2.473979
SYT4	synaptotagmin 4 [Homo sapiens (human)]	3.03E-07	2.436962
STK32B	serine/threonine kinase 32B [Homo sapiens (human)]	1.69E-05	2.389932
KIAA1377	centrosomal protein 126	3.73E-03	2.384472
ENST00000390632	immunoglobulin heavy variable 3–66	1.32E-04	2.355071
HOXA11	homeobox A11 [Homo sapiens (human)]	4.57E-07	2.319228
ATF7IP2	activating transcription factor 7 interacting protein 2 [Homo sapiens (human)]	2.59E-05	2.313631
MACC1	MACC1, MET transcriptional regulator [Homo sapiens (human)]	2.21E-02	1.516496

**Table 3:** Deregulated genes in CD133^lo^ CSCs when compared to CD133^hi^ non-CSCs. List of top 30 genes down-regulated in CD133^lo^ cells

Gene ID	Gene Name	PValueLogRatio	MEAN_LOG2_R\G_RATIO
A_24_P938577	follicular lymphoma variant translocation 1	2.31E-04	−2.61517
A_24_P911519	unknown	4.63E-05	−2.62426
C12orf12	coiled-coil glutamate rich protein 1 [Homo sapiens (human)]	3.55E-14	−2.62559
A_24_P942870	unknown	1.29E-02	−2.68738
TSHR	thyroid stimulating hormone receptor [Homo sapiens (human)]	4.27E-02	−2.68954
A_24_P800363	AU146536 HEMBB1 Homo sapiens cDNA clone HEMBB1000770	2.28E-01	−2.74867
CDH6	cadherin 6 [Homo sapiens (human)]	5.85E-02	−2.74995
A_24_P376029	A_24_P376029	1.00E + 00	−2.77371
GFM1	G elongation factor mitochondrial 1 [Homo sapiens (human)]	6.18E-02	−2.79011
A_24_P642426	a_24_P376029	5.80E-02	−2.80546
A_32_P849727	A_24_P642426	1.00E + 00	−2.80911
DLX2	distal-less homeobox 2 [Homo sapiens (human)]	2.77E-16	−2.8165
A_24_P931377	A_32_P849727	1.56E-18	−2.8605
ROR1	receptor tyrosine kinase like orphan receptor 1 [Homo sapiens (human)]	1.17E-06	−2.86722
TFEC	transcription factor EC [Homo sapiens (human)]	3.05E-02	−2.87202
MITF	melanogenesis associated transcription factor [Homo sapiens (human)]	4.87E-02	−2.87412
ENST00000328752		2.47E-06	−2.91985
A_24_P923789	A_24_P923789	2.10E-02	−2.92733
KNG1	kininogen 1 [Homo sapiens (human)]	1.33E-04	−2.96233
SCN9A	sodium voltage-gated channel alpha subunit 9 [Homo sapiens (human)]	1.00E + 00	−2.99605
ENST00000436580		1.00E + 00	−3.11519
LMOD3	leiomodin 3 [Homo sapiens (human)]	1.00E + 00	−3.13762
A_24_P923439	A_24_P923439	9.65E-11	−3.14399
ENST00000481704		4.02E-06	−3.15966
FGF5	fibroblast growth factor 5 [Homo sapiens (human)]	3.19E-01	−3.25497
A_32_P53093		3.03E-01	−3.45255
CCDC68	coiled-coil domain containing 68 [Homo sapiens (human)]	2.63E-20	−3.50641
ENST00000354854		2.72E-06	−3.516
CXorf57	chromosome X open reading frame 57 [Homo sapiens (human)]	6.69E-03	−3.5772
AK000119		9.79E-04	−3.67492

**Table 4:** Deregulated genes in CD133^lo^ CSCs when compared to CD133^hi^ non-CSCs. Embryonic and Neural stem cell markers up-regulated in CD133^lo^ cells

Genes highly expressed in CD133lo cells	Gene Name	PValueLogRatio	Fold change
HOXB2	homeobox B2	4.67E-02	2.4
HOXA9	homeobox A9	1.77E-06	2.3
SALL1	spalt like transcription factor 1	4.35E-07	2.2
ABCB1	ATP binding cassette subfamily B member 1	5.60E-04	2.2
ABCB5	ATP binding cassette subfamily B member 5	1.24E-01	2.1
LEFTY	left-right determination factor	7.52E-02	2.0
MUSHASHI 2	musashi RNA binding protein 2	1.97E-01	1.6
BMI-1	BMI1 proto-oncogene, polycomb ring finger	8.04E-04	1.6

## Discussion

Retinoblastoma is a small round cell tumor that comprises of rapidly dividing tumor cells, arising from the retina, within extensive areas of ischemic necrosis as they outgrow their own blood supply [25]. This study attempts to characterize the stem-like cancer cells in the well-established Rb Y79 cell line using the surface marker widely used in other tumors for CSC isolation- CD133 (Prominin). The study confirms that Y79 Rb cell line harbours stem-like cancer cells endowed within the CD133^lo^ enriched population. This was demonstrated by CSC properties such as exclusive surface marker phenotype, size and percentage, slow cycling/dormancy, clone forming ability and differentiation, invasiveness, chemoresistance and primitive gene expression markers.

While in several solid tumors [26], CD133^hi^ cells are heralded as the cells with CSC properties, there are also contrasting reports of CD133^lo^ subset being capable of exhibiting tumorigenicity and clone forming ability in tumors such as Glioma [27], Glioblastoma [28], Colon cancer [29], etc. Sun and co-workers reported that in neural stem cells, the CD133 negative population are clonogenic and slow cycling in nature [30]. In the developing retina, CD133, which is a conserved surface glycoprotein, is reported to be acquired upon differentiation. In support of this observation is the study by Lakowski et al. who reported that the neuroblastic layer of developing photoreceptors showed lower expression of CD133 when compared to the outer segments of the photoreceptors that had intense expression [19]. Also, Xu and their group showed that in Rb deficient retinal cells, CD133 expression was strong in maturing photoreceptors and weak in the retinal progenitor cell population [20]. Studies have shown that CD133 (Prominin) is crucial for photoreceptor outer segment morphogenesis and the mutations within the gene is associated with several retinal dystrophies which ascertains its pivotal role in the visual cycle [31]. It therefore appears logical to speculate that the CD133 is a marker acquired after cellular differentiation in Retinoblastoma, and the putative CSCs would lack the expression similar to progenitors, since they are believed to arise from the undifferentiated retinal cells.

One of the challenging issues in characterizing CSCs is the sorting/enrichment strategies which are crucial for validating the functional attributes of stem-like cancer cells [22].With regard to Rb primary tumors, our earlier work highlighted, using a bimodal pattern, that FSC^lo^/SSC^lo^ CD133^lo^/CD44^hi^ cells show more primitive markers as compared to CD133^hi^ cells [18]. In contrast to our observation of CD133^lo^ cells as the Rb CSC subset, Hu et al. using the principle of generating stem-like cancer cells by serum-free culture in WERI-Rb cell line, documented higher percentage of CD133^hi^ cells within the clones when compared to cultured WERI-Rb cells [15]. Based on the evidence of varying expression of CD133 expression in different stages of embryonic stem cell development and its differentiation into neural lineage [32], it is logical to speculate that there could be a range of expression within tumors of different lineages. Similarly, it could also be affected by modifications induced in primary tumors and the culture conditions of cell lines, which needs to be explored further. This interesting and contrasting expression possibly reflects the evolving concept that the CSC properties of tumor cells enhance or decrease in a gradual fashion and is possibly tissue specific. The surface markers CD90 and CXCR4 are within the range reported by our earlier study in patient samples and correspond to 79.7 ± 1.3% and 14.4 ± 0.5% respectively. This study confirms the presence of CSCs in Y79 cell line comparable to the surface markers observed in the primary tumors [18], however with one major difference which is the absence of CD44 expression in all cells. Lack of expression of CD44 (Hyaluronic acid receptor) could possibly be attributed to culture conditions and absence of hyaluronic acid, which is abundantly present in the vitreous fluid [33]. This observation is also supported by Ma et al.*,* who showed that long-term serum-free cultures of neurospheres from primary Retinoblastoma showed increased expression of CD44 marker in addition to CD133 when compared to the in vitro differentiated cells [21]. The evidence from retinal studies and increasing expression of CD133 in long-term cultures of Rb supports our observation of CD133 as a marker of differentiation. A thorough comparison of CSC functional properties between the CD133 enriched populations, that was lacking in earlier reports, is highlighted in this study which ascertains the robustness of CD133^lo^ phenotype as a CSC marker.

Another important characteristic of CSCs appears to be the dormancy of cells and segregation within the G0/G1 phase of the cell cycle indicating that they are inherently slow cycling. It has been reported that CSCs are quiescent/slow cycling in nature unless triggered by selection pressure [34]. While the bulk of the tumor population remains in a constant balance between the G_1_/G_2_-S-M phases, CSCs are believed to remain in a G_0_/G_1_ phase [35]. This attribute makes the CSCs resistant to conventional therapies that target the rapidly dividing tumor cells. In ovarian cancer, Kusumbe et al. showed that the quiescent fraction of the tumor exhibited stem cell activity and ability to revert to a state of self-renewal and differentiation [36]. Our study also demonstrated that CD133^lo^ cells were observed to be in the G_0_/G_1_ phase implicating that these are the slow cycling and dormant cells.

The ability to form large colonies within a short period is another hallmark of stem-like cancer cells. Using the soft agar colony forming assay, the study documented that the CD133^lo^ cells were capable of forming larger colonies in soft agar when compared to smaller colonies of the CD133^hi^ subset in 2 weeks of culture. Siegel et al. has shown the evidence for neurosphere forming ability of Y79 cells in culture for 5 days [13]. However, clone forming ability of CD133 sorted populations is one of the novel features of this study. The experiments revealed CD133^lo^ cells formed increased number of large sized colonies as against CD133^hi^ cells in an anchorage independent colony formation assay. This is in concordance with the defined characteristics of holoclones and paraclones in human epidermal keratinocytes by Barrandon and Green and the more recent study on prostate cancer cell clones by Beaver et al. [37, 38]. The CD133^lo^ cells form large clusters of small sized cells with a smooth outline representative of holoclones, whereas CD133^hi^ cells form much smaller colonies with irregular margins depicting paraclones. CD133^lo^ colonies exhibited larger colony area than the CD133^hi^ clusters after 2 weeks hinting at their ability to self-renew and proliferate. The CD133^lo^ population with higher clonogenicity further supports the CSC nature of these cells. The clones could be expanded to passage 5 and showed gradual increase in CD133^hi^ cells with increasing passages suggesting that these cells are capable of self-renewal as well as differentiation to CD133^hi^ cells. In support of this observation is a study by Wang et al. whose group reported that glioma cells that were CD133 negative were capable of forming tumors in nude rats and differentiating into CD133 positive cells [27]. The CSCs generated CD133^hi^ cells upon multiple passages thereby confirming both self-renewal and differentiation. The results from these functional studies of CD133^lo^ vs CD133^hi^ Y79 cells strongly hint that CD133 is a marker of differentiated/mature Rb tumor cells. Validation of this by in vivo tumorigenicity assays would be a valuable study in future.

The invasive potential of these CSCs were evaluated using a matrigel transwell system and the serum starved CD133^lo^ cells exhibited higher invasiveness (*p* < 0.05) when compared to their counterparts. These observations are in agreement with other tumors such as breast cancer, colon cancer, ovarian cancer, etc., in which the CSCs were shown to have increased metastatic potential which concurs with our findings [39–41].

The CD133^lo^ cells also exhibited chemoresistance towards Carboplatin at higher doses (*p* < 0.001), which caused cytotoxicity in the CD133^hi^ population at the end of 48 h exposure. CSCs have shown to have increased chemoresistance which helps them to overcome the therapeutic killing by being able to efflux drugs and resist cell death [42, 43]. In the CD133^lo^ population, it was noted that these cells were not only capable of resisting cell death but also exhibited high proliferation in presence of Carboplatin at the end of a 48 h exposure. On the contrary, CD133^hi^ cells showed a profile similar to the total Y79 population with decreasing viability upon exposure to increasing drug concentrations. It is possible to extrapolate that this could be one of the reasons why in some of the cases of Retinoblastoma, the enucleated eyeballs from patients who have received chemotherapy, showed evidence of viable tumor cells with mitotic activity [44].

Gene expression studies in Y79 cell line revealed several pathways deregulated in the putative CSCs with significant up-regulation of stem cell genes and genes involved in proliferation, chemoresistance and metastasis when compared to the non-CSC population. In support of the evidence in primary tumor cells [18], where CD44^hi^CD133^lo^ cells exhibited the progenitor cell markers, the CD133^lo^ cells showed similar marker profile except for CD44 expression. Microarray data also shows that there was significant higher expression of embryonic and neural stem cell markers in the stem-like cancer cells of Y79 cell line. Stem and progenitor cell genes such as *OCT4*, *NANOG*, *BMI1*, *ABCG2* and *PROX1* were highly expressed in CD133^lo^ population. This is in concordance with the gene expression findings by few groups on total and ABCG2 enriched Rb Y79 cells, and WERI-Rb cells [13, 15, 16]. This study also identified an important regulator of metastasis, Metastasis-associated in colon cancer 1 (*MACC1*) gene to be expressed in Y79 cell line. To the best of our knowledge, this is the first report of *MACC1* overexpression in Retinoblastoma and specifically within the CD133^lo^ subset of Y79 cell line. This gene has been recently identified as an important factor in tumor cell proliferation, invasion and metastasis in several cancers such as Colon, HCC, Breast, Ovarian, etc. via the c-MET/HGF signalling pathway and is being evaluated as a potential therapeutic target [45, 46].

This study also has its set of limitations, the foremost being lack of a positive co-marker for CSCs. Even though the functional studies validate the CD133^lo^ Y79 cells being endowed with CSC properties, which are in tune with the findings in primary tumors; positive co-markers would add more strength to the data. The multidrug resistance markers that were identified in the microarray study (*ABCB1*, *ABCB5*) could be used for co-localization studies in future. Though quiescence is one of the hallmarks of CSCs, documentation of the same is a challenge. Cell cycle analysis does not segregate the G0/G1 phases or the different population of cells within the G0 phase of cell cycle, which could also contain differentiated, senescent and stem cells. The study partly addressed it using surface phenotype and clone forming ability, however, segregating the different populations in G0/G1 phase would add value to the study. The CFE measurement was performed for the colonies formed at a 2-week period. Longer incubation period and characterization of the primary clones could further ascertain the clone forming ability and stem cell phenotype. However, the cumulative evidence of all the investigations point towards the CD133^lo^ cells being endowed with CSC properties in Rb Y79 cell line which is a novel finding of this study. We believe that similar CSC characterization studies on other Rb cell lines and primary cells would add more strength to the hypothesis.

## Conclusions

This study confirms that the CD133^lo^ subset of Rb Y79 cell line is endowed with the characteristics of cancer stem cells as demonstrated by the in vitro functional assays and is in agreement with our previous findings in primary Rb tumors. This insight of tumor heterogeneity and hierarchy is quite vital for designing better strategies towards a successful therapeutic outcome.

## Abbreviations

ANOVA: Analysis of variance
APC: Allophycocyanin
BrdU: 5-bromo-2′-deoxyuridine
cDNA: Complementary DNA
CFE: Colony forming efficiency
C-MET/HGF: hepatocyte growth factor receptor/hepatocyte growth factor
CSC: Cancer stem cells
CTP: Cytidine triphosphate
Cy3/Cy5: Cyanine 3/5 dye
DAVID: Database for annotation, visualization and integrated discovery
DMSO: Dimethyl sulfoxide
ELISA: Enzyme-linked immunosorbent assay
FBS: Fetal Bovine Serum
FSC: Forward scatter
GLP: Good laboratory practices
HCC: Hepatocellular carcinoma
KEGG: Kyoto encyclopaedia of genes and genomes
MTT: 3-(4,5-Dimethylthiazol-2-yl)-2,5-Diphenyltetrazolium Bromide
PBS: Phosphate-buffered saline
PCR: Polymerase chain reaction
PFA: Paraformaldehyde
Rb: Retinoblastoma
RNA: Ribonucleic acid
RPMI: Roswell Park Memorial Institute-1640
SEM: Standard error of the mean
SLCC: Stem-like cancer cells
SSC: Side scatter
SV40 βLH-T-Ag: simian virus-40 luteinising hormone β sub-unit Large T-antigen
TGF-B: Transforming growth factor beta
TIC: Tumor initiating cells

## GENES

ABCB1: *Homo sapiens* ATP binding cassette subfamily B member 1
ABCB5: *Homo sapiens* ATP binding cassette subfamily B member 15
ABCG2: *Homo sapiens* ATP binding cassette subfamily G member 2
ACTB: *Homo sapiens* actin beta
ALDH1: *Homo sapiens* aldehyde dehydrogenase 1 family member A1
BMI1: *Homo sapiens* BMI1 proto-oncogene, polycomb ring finger
CD133: *Homo sapiens* prominin 1
CD44: *Homo sapiens* CD44 molecule
CXCR4: *Homo sapiens* C-X-C motif chemokine receptor 4
HOXA9: *Homo sapiens* homeobox A9
HOXB2: *Homo sapiens* homeobox B2
LEFTY: *Homo sapiens* left-right determination factor
MACC1: *Homo sapiens* metastasis associated in colon cancer 1
MELK: *Homo sapiens* maternal embryonic leucine zipper kinase
MSI1: *Homo sapiens* musashi RNA binding protein 1
MSI2: *Homo sapiens* musashi RNA binding protein 2
MYCN: *Homo sapiens* proto-oncogene, bHLH transcription factor
NANOG: *Homo sapiens* Nanog homeobox
NESTIN: *Homo sapiens* nestin
OCT4: *Homo sapiens* POU class 5 homeobox 1
PROX1: *Homo sapiens* prospero homeobox 1
RB1: *Homo sapiens* RB transcriptional corepressor 1
SALL1: *Homo sapiens* spalt like transcription factor 1
SNAI2: *Homo sapiens* snail family transcriptional repressor 2
SOX2: *Homo sapiens* SRY (sex determining region Y)-box 2
SYX1A: *Homo sapiens* Syntaxin 1A
